# CNN and LSTM-Based Emotion Charting Using Physiological Signals

**DOI:** 10.3390/s20164551

**Published:** 2020-08-14

**Authors:** Muhammad Najam Dar, Muhammad Usman Akram, Sajid Gul Khawaja, Amit N. Pujari

**Affiliations:** 1 Department of Computer and Software Engineering, College of Electrical and Mechanical Engineering, National University of Sciences and Technology, Islamabad 44000, Pakistan; usman.akram@ceme.nust.edu.pk (M.U.A.); sajid.gul@ceme.nust.edu.pk (S.G.K.); 2 School of Engineering and Technology, University of Hertfordshire, Hatfield AL10 9AB, England, UK; amit.pujari@ieee.org; 3 School of Engineering, University of Aberdeen, Aberdeen AB24 3UE, Scotland, UK

**Keywords:** convolutional neural network (CNN), long short-term memory (LSTM), emotion recognition, EEG, ECG, GSR, deep neural network, physiological signals

## Abstract

Novel trends in affective computing are based on reliable sources of physiological signals such as Electroencephalogram (EEG), Electrocardiogram (ECG), and Galvanic Skin Response (GSR). The use of these signals provides challenges of performance improvement within a broader set of emotion classes in a less constrained real-world environment. To overcome these challenges, we propose a computational framework of 2D Convolutional Neural Network (CNN) architecture for the arrangement of 14 channels of EEG, and a combination of Long Short-Term Memory (LSTM) and 1D-CNN architecture for ECG and GSR. Our approach is subject-independent and incorporates two publicly available datasets of DREAMER and AMIGOS with low-cost, wearable sensors to extract physiological signals suitable for real-world environments. The results outperform state-of-the-art approaches for classification into four classes, namely High Valence—High Arousal, High Valence—Low Arousal, Low Valence—High Arousal, and Low Valence—Low Arousal. Emotion elicitation average accuracy of 98.73% is achieved with ECG right-channel modality, 76.65% with EEG modality, and 63.67% with GSR modality for AMIGOS. The overall highest accuracy of 99.0% for the AMIGOS dataset and 90.8% for the DREAMER dataset is achieved with multi-modal fusion. A strong correlation between spectral- and hidden-layer feature analysis with classification performance suggests the efficacy of the proposed method for significant feature extraction and higher emotion elicitation performance to a broader context for less constrained environments.

## 1. Introduction

Recent trends in the field of affective computing have shifted towards a more reliable source of physiological signals [1,2,3,4] such as Electroencephalogram (EEG), Electrocardiogram (ECG), and Galvanic Skin Response (GSR) due to their significance in human–computer interaction (HCI). Emotions can be distinctively expressed as a non-verbal form of everyday social interaction. These non-verbal cues are generally reflected through facial expressions and tone of voice. However involuntary physiological responses (such as EEG and ECG) to the emotional stimuli are more reliable compared to voluntary response (such as sound or facial expressions), because the involuntary response cannot be masked intentionally [4] (a sad person may smile, which may also be the indication of depression). Similarly, external factors such as lighting conditions, accessories like glasses or a hat, and surrounding audio noise may affect the performance of these external voluntary modalities of expression, limiting their use in computing applications [5]. Biosensors help to monitor and collect physiological signals from heart (ECG), brain (EEG), or skin (GSR), and proves to be the most significant for the detection of stress levels and emotions [6,7]. Applications of physiological signal-based emotion recognition encompass psychological health-care monitoring for hospitalized patients [8], real-time stress-level detection of drivers, emotion-inspired multimedia applications [9], various bio-inspired human–machine interfaces, and health-care applications [10].

**Figure 1:**
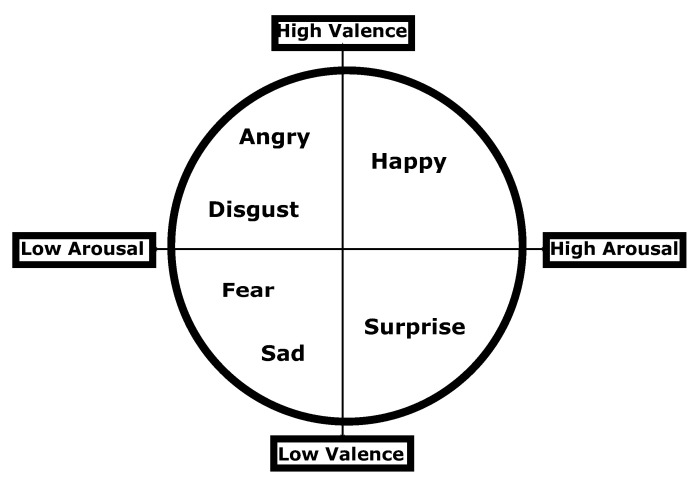
Valence arousal model for emotion elicitation.

The problem of emotion elicitation is generally approached in the literature by measuring valence and arousal as represented in the emotion circumplex model [11]. A simple representation of the circumplex model is shown in Figure 1, which exhibits the positions of six basic emotions (happiness, anger, disgust, fear, sadness, and surprise) on the scales of valence and arousal. Valence expresses the positivity or negativity of the emotion; arousal expresses the degree of excitement covered by the emotion stimuli. Most of the affective computing literature based on physiological signals classifies valence (high/low) and arousal (high/low) as separate binary classification tasks [12,13]. However, little other research has approached the problem in the broader context of the classification of emotions into four categories [14,15] of High Valence—High Arousal (HVHA), High Valence—Low Arousal (HVLA), Low Valence—High Arousal (LVHA) and Low Valence—Low Arousal (LVLA).

The process of emotion elicitation in the literature generally revolves around conventional feature extraction from physiological signals. For EEG-based classification, the conventional statistical features, wavelet features, and Empirical Mode Decomposition (EMD)-based features were applied to test the efficacy of these algorithms for emotion elicitation. For instance, Support Vector Machine (SVM) was tested with the efficacy of EMD in combination with the genetic algorithm [16]. Stable statistical features such as band power, coherence, and entropy were proposed [17] for the classification of four emotions, namely happiness, fear, pleasure, and anger. Similar conventional approaches for ECG-based emotion recognition were also applied in the literature. In [18], time-domain ECG features after computation of P-QRS-T wave detection were extracted and selected through hybrid Particle Swarm Optimization (PSO) with a Fisher classifier for emotion recognition. However, these approaches used their private datasets for emotion classification. The more standard approaches incorporated publicly available datasets of DEAP for EEG and the MAHNOB-HCI dataset for ECG. For instance, relative wavelet energy with SVM was used in [19] for the DEAP dataset, with the recognition rate of 61.8%. SVM classification was proposed [20] for the wavelet-based extracted EEG features from the DEAP dataset to achieve 65.13% and 65.33% accuracy for binary classification of valence and arousal, respectively. One of the approaches [2] for ECG signals from the MAHNOB-HCI dataset was based on neighborhood component analysis for binary classification of valence and arousal. In [21], heart rate variability-based statistical features were incorporated with SVM for the recognition of five emotions using the MAHNOB-HCI dataset.

Novel trends in the computation of emotion elicitation exploit Deep Neural Networks (DNN) for physiological signals with improvement in recognition rates. One of the earliest attempts was made by [22], using Wavelet Transform and Back-Propagation Neural Networks (BPNN)-based emotion recognition from Electromyogram (EMG) signals to recognize four emotions of joy, sadness, anger, and pleasure. Another early attempt proposed [23] Radial Basis Function (RBF) Neural Network for an emotion elicitation task. However, both approaches used private datasets for the computation of emotions; the publicly available dataset of DEAP is also incorporated using DNN architectures. Long Short-Term Memory (LSTM) was deployed [24] for EEG-based emotion elicitation and reported recognition rates of 72.06% and 74.12% for binary classification of valence and arousal using DEAP dataset. A more recent study [25] proposed Graph-regularized Extreme Learning Machine (GELM) for the classification of HVHA, HVLA, LVHA, and LVLA. They incorporated spectral features from EEG with the GELM classifier for SEED [26] and DEAP datasets, respectively. In [27], LSTM networks were incorporated in 5sec segments of EEG signal from the DEAP dataset to report recognition rates of 85.45% and 85.65% for binary classification of valence and arousal, respectively. Similarly, Ref. [28] claimed the significance of deep CNN architecture for EEG-based emotion recognition using the DEAP dataset. The authors reported a recognition rate of 73.36% and 81.4% for binary classification of arousal and valence, respectively. Recognition results obtained from conventional and DNN-based approaches as described in [20,24,27] point towards the significance of DNN-based algorithms trends as compared to conventional approaches.

Various modalities such as face videos, eye movement, ECG, EEG, and GSR, etc. were incorporated in the literature for emotion elicitation. A multi-modal approach combines any of these modalities for better recognition performance. DNN-based multi-modal approach in the literature generally works in two dimensions—one of the dimension involves the joint representation of multi-modality with feature-level fusion, and the other dimension involves the coordinated representation of multi-modality with decision-level fusion [29]. Feature-level fusion concatenates sets of extracted features from various modalities before the classification learning stage, while decision-level fusion merges the classification decisions of various classifiers or deep networks for specific modalities [30]. In [31], feature-level fusion emotion recognition is applied using physiological signals. This study incorporated the decision-level fusion scheme of majority voting. In [32], the authors proposed multi-layer LSTM for the multi-modal fusion of EEG signals with videos for binary classification of valence and arousal respectively for MAHNOB-HCI dataset. In [33], eye movement and EEG-based decision-level multi-modal fusion was applied by analyzing confusion matrices. The significance of decision-level fusion lies in the accessibility of modality comparison in terms of its overall contribution to the recognition results [29]. In our study, we incorporate decision-level fusion based on majority voting due to the simplicity of the procedure for the combination of modalities.

Based on the previous research examination, there are the following few things that need to be addressed for the contribution of existing knowledge. ECG is less explored in the literature as compared to EEG, despite its higher significance in emotion elicitation. There is a need to build a high-performance non-invasive emotion recognition process using low-cost wearable sensors for broader classes of emotions. Connectivity among spectral features and the features extracted by deep neural networks need to be examined to understand and build more robust neural network architectures specific to the spectral properties of modality. EEG, ECG, and GSR are continuous-time signals with high memory content, which could be exploited by LSTM for better results. LSTM has the property of selectively remembering patterns for a long duration of time useful in feature extraction from physiological signals. Due to the irregular structure of EEG [13], 14 channels need to be arranged in the 2D image to exploit the usefulness of a 2D Convolutional Neural Network (CNN). Therefore, the proposed 1D-CNN + LSTM architecture is used to exploit their usefulness for time-series data of ECG and GSR as well as 2D-CNN architecture for image representation of 14-channel EEG. We selected recently published, publicly available datasets of AMIGOS [34] and DREAMER [35] for the emotion recognition in less constrained scenarios with the additional ensemble of modalities at the decision level.

The rest of the paper is organized as follows. Section 2 examines the state-of-the-art literature in detail. It also exposes research gaps identified as the implication of previous research and our contribution to fill those gaps in Section 2.1 and Section 2.2. The details of the datasets used in this study and the details of proposed methodology are discussed in Section 3. Section 4 provides results on both datasets, and detailed discussion and spectral analysis of results are provided in Section 5. The discussion section also includes the detailed analysis and representation of deep features from hidden layers of proposed DNN architecture. Conclusions drawn from this research are finally presented in Section 6.

## 2. Related Work

To establish a comparison of our computational framework with related works, most relevant studies based on AMIGOS and DREAMER datasets are described in this section. A recent study [36] proposed a fusion of statistical features extracted from EEG, ECG, and GSR from the AMIGOS dataset. They reported a recognition rate of 67% and 68.8% for valence and arousal respectively using an SVM classifier. Another recent GSR-based framework [37] using AMIGOS dataset proposed temporal and spectral features with SVM (RBF kernel) to report recognition performance of 83.9% and 65% for valence and arousal, respectively. For the AMIGOS dataset, the significance of DNN can be explained by two similar studies, where one of the studies [34] reported 55.1% and 54.4% F1 scores for valence and arousal, respectively, using Gaussian Naive Bayes, while another study [38] reported 71% and 81% accuracy for valence and arousal, respectively, using convolutional neural networks.

In a recent study [39], the authors proposed self-supervised learning instead of self-assessment labels for the AMIGOS dataset using a convolutional neural network. They reported a recognition rate of 84% and 85.8% for valence and arousal, respectively. LSTM-RNN is recently proposed [40], with the use of attention-based mechanism for AMIGOS dataset, and reported recognition rates of 79.4% and 83.3% for binary classification of valence and arousal, respectively. In [14], 3D-CNN and 1D-CNN models are compared and report the accuracy of 99.7% for four classes of emotion. The significance of results for four classes of emotion can be understood using the recent two studies. A study [41] reported an accuracy of 66.67% and 71.54% for binary classification of valence and arousal, respectively, and reported 38.28% classification accuracy for four classes (HVHA, HVLA, LVHA, and LVLA) of emotions using the same proposed methodology. Another similar recent study [15] reported accuracy of 83.02% and 82.74% for binary classification of valence and arousal respectively, while reported 58.57% classification accuracy for four classes (HVHA, HVLA, LVHA, and LVLA) of emotions.

Currently using the DREAMER dataset, Graph Convolutional Broad Network (GCB-net) is suggested [13] to announce an accuracy of 86.99% and 89.32% for binary classification of valence and arousal respectively. In [12], using DREAMER, the accuracy of 86.23% and 84.54% are reported for classification of valence and arousal, respectively. In [35], a multi-modal fusion of EEG and ECG of the DREAMER dataset was incorporated with 61.84% and 63.32% recognition rates for valence and arousal, respectively.

To evaluate studies using both the DREAMER and AMIGOS dataset, Ref. [15] reported an accuracy of 79.95% for binary classification of both valence and arousal using the DREAMER dataset. They also reported accuracy of 83.94% and 82.76% for binary classification of valence and arousal respectively for the multi-modal fusion of physiological signals in the AMIGOS dataset. The most recent study [42] based on the AMIGOS dataset proposed the Bayesian network to achieve accuracy of 90% for binary classification of the high and low level of valence. They also reported accuracy of 86% for binary classification of the high and low level of valence for the DREAMER dataset.

### 2.1. Implications

It is interesting to state a few implications here from an extensive literature review based on a physiological signal-based emotion recognition. First, the deep-learning approaches especially convolutional and LSTM-based neural networks performed much better in terms of recognition performance of emotions as compared to conventional feature extraction and simple classifier-based approaches [9]. Second, EEG is extensively used for the emotion elicitation process; however, the separate significance of ECG and GSR modalities in terms of long duration sequence data are much less explored for this purpose especially using DNN-based approaches [43]. Third, most of the studies in the literature [44,45] with the most promising results are based on the evaluation of binary classification of valence (high valence, low valence) and arousal (high arousal, low arousal) separately. However, in the case of four classes of emotions (HVHA, HVLA, LVHA, and LVLA) the reported recognition rates decrease to a much larger extent [17]. Fourth, due to the highly subjective and dynamic nature of emotion, few pieces of research have proposed subject-dependent approaches with better recognition performance; however, subject-dependent approaches besides higher recognition rates require large training data from each subject and are not capable of being applied to unseen subjects [46]. Therefore, studies reported for subject-independent approaches [47,48] are more reliable for emotion elicitation tasks from a broader perspective as compared to the results obtained using subject-dependent approaches [49,50]. Fifth, recognition performance of emotion is more reliable and comparable when reported for publicly available datasets acquired using portable and wearable sensors [34,35] for their use in real-world environments as compared to invasive and restricted lab environments.

### 2.2. Our Contribution

We proposed the combination of LSTM and CNN architecture to improve the recognition performance for four classes (HVHA, HVLA, LVHA, and LVLA) of emotion with multi-modal fusion while exploiting the significance of various modalities such as ECG, EEG, and GSR. Our approach is subject-independent and recognition rates are reported against two of the publicly available datasets of AMIGOS and DREAMER. Both datasets acquired signals using wearable, low-cost sensors to represent the significance and reliability of proposed methodology in real-world environments.

## 3. Material and Methods

This section will elaborate the significance and details of datasets used in this study. After elaboration of materials used, proposed methodology will be discussed in detail.

### 3.1. Datasets

Two of the publicly available datasets named DREAMER and AMIGOS were incorporated for the evaluation of proposed deep neural network architectures. Both of these datasets are newly published yet extensively used in recent literature for physiological signal-based emotion elicitation, primarily because of their non-invasive nature of the data acquisition process using low-cost off-the-shelf devices. In both of the datasets, a 14-channel Emotiv Epoc [51] wireless headset for EEG and two-channel Shimmer ECG sensor [52] was used as compared to quite an invasive headset of Biosemi active two [53] used in DEAP [54] and MAHNOB-HCI [55] datasets. Biosemi active two is much more accurate and precise, and leverages more channels for EEG collection; however, invasive data acquisition procedure is required using this device. The DREAMER and AMIGOS dataset helps to improve emotion classification performance for the non-invasive environment with low-cost sensors. DREAMER data is provided with raw ECG and EEG signals, therefore dedicated basic pre-processing steps were applied in Section 3.3.1 to raw DREAMER dataset to be consistent with the basic pre-processed signals available for AMIGOS dataset. The concise details of both datasets are given below.

#### 3.1.1. DREAMER

This dataset used audio-visual stimuli for affect elicitation from 23 subjects (14 males and 9 females). Each subject was exposed to 18 different trials of variable length from 65 s–393 s duration with the addition of a 61 s baseline signal (where there was no stimulus provided as a neutral state) for each trial. Each subject was asked to label each trial with the valence and arousal values for the scale of 1–5 using Self-Assessment Manikins (SAM). 14 channels of EEG with AF3, F7, F3, FC5, T7, P7, O1, O2, P8, T8, FC6, F4, F8, AF4 channels were recorded using Emotiv Epoc portable sensor with the sampling frequency of 128 Hz. ECG with two channels was recorded using the Shimmer sensor with a sampling rate of 256 Hz. 57 s of baseline signals (segmenting out the first two and last two seconds) and the last 60 s of each stimulus trial signals is incorporated for this study.

#### 3.1.2. AMIGOS

The AMIGOS dataset also acquires 14-channel EEG (same channel position as described for DREAMER) and two ECG channels using similar sensors and sampling frequency used for the DREAMER dataset. However, the AMIGOS dataset used audio-visual stimuli of 16 different short-term trails with a variable duration from 51 s–150 s and the addition of a 5 s baseline (without stimulus) for each trial. The signals were self-annotated (using SAM) for each trial with the valence and arousal values on the scale of 1–9. Basic pre-processed data from AMIGOS is used for the complete length of trials and 5 s of baseline for 33 subjects with valid data out of total 40 subjects with the exemption of 7 subjects (with ID number 33, 24, 23, 22, 21, 12, 9) with invalid data. DREAMER published a dataset of 23 out of 25 persons with valid data only and exempted 2 persons with invalid data, therefore all the 23 person data from the DREAMER dataset is used. AMIGOS dataset in comparison to DREAMER additionally provided with GSR data, which is also incorporated in this study for comparison.

### 3.2. Methodology

**Figure 2:**
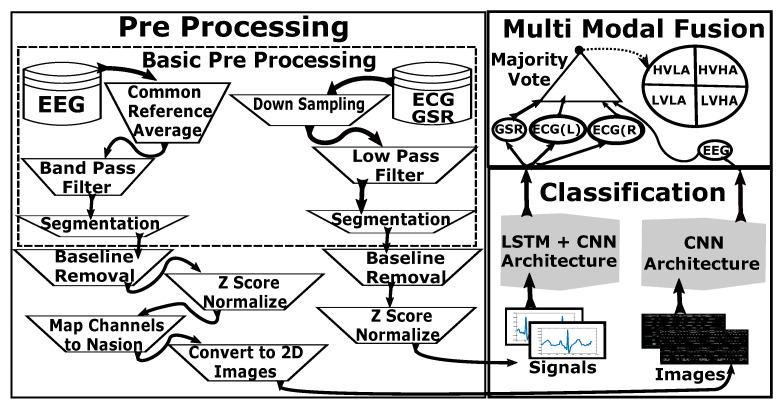
Block Diagram of complete methodology.

The three main components of the proposed algorithm are pre-processing, classification, and multi-modal fusion. First, all physiological signals are required to be pre-processed before their use in neural network architecture [56]. EEG is quite different from other peripheral signals in terms of the number of channels, frequency, and amplitude. Therefore, the sequence of steps applied to the EEG is generally represented in parallel to the sequence of steps applied for other modalities before multi-modal fusion. Figure 2 represents a general block diagram of all the steps applied in this study. The following subsections represent a detailed description of these steps applied in the proposed methodology.

### 3.3. Pre-Processing

Pre-processing steps are divided into two categories, namely basic pre-processing and specialized pre-processing. The basic pre-processing steps are common to the generic applications of physiological signals. The basic pre-processing steps were followed by the steps used in basic pre-processing of AMIGOS dataset [34] to standardize physiological signals from both datasets. These steps involve signal filtering and noise removal, and are common for both GSR and ECG signals. However, EEG signals required different pre-processing steps as compared to ECG and GSR due to different inherent properties of EEG [57]. The second category with specialized pre-processing consists of steps required specific to the emotion elicitation algorithm. These steps include baseline removal and Z-score normalization, inspired by its significance in many studies such as [46,58,59,60,61].

#### 3.3.1. Basic EEG Pre-Processing

The Basic EEG pre-processing framework consists of referencing common average, band-pass filtering, and segmentation. First, raw EEG signals are required to be re-referenced to remove channel biases introduced by online reference [62]. These channel biases affect the amplitude of EEG channels based on their spatial locality from online reference. The common average was computed by extracting the average EEG signal of all the 14 EEG channels as shown in Equation (1), where *c* represents the channel number from 1 to N=14 and *l* is the length of EEG channel (64 s with the total of 8192 samples) each for DREAMER dataset.

(1) $$meanEEG_{l}=\frac{1}{N}\left(\sum_{c=1}^{N}EEG_{c,l}\right)$$

(2) $$acrEEG_{c,l}=EEG_{c,l}-meanEEG_{l}$$

**Figure 3:**
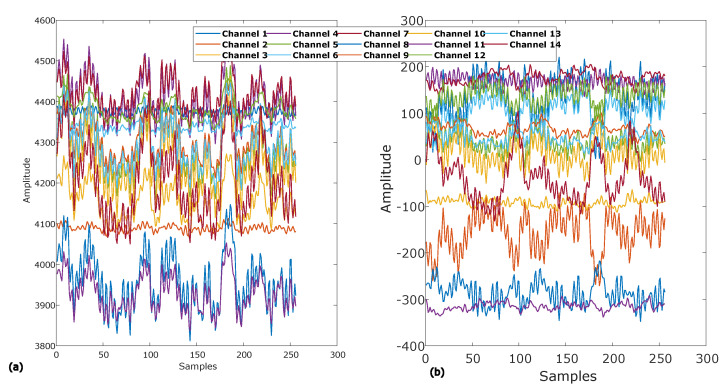
(**a**) Raw EEG signals of 14 channels. (**b**) EEG Signals of 14 channels averaged to common reference.

Now each of the 14 EEG channels with length l are subtracted from the computed common average as described in Equation (2) to obtain EEG signals with averaged to a common reference. The results of basic EEG pre-processing are depicted in Figure 3. Figure 3a represents raw EEG signals of all the 14 channels and Figure 3b represents all channels of EEG signals after averaged to a common reference.

**Figure 4:**
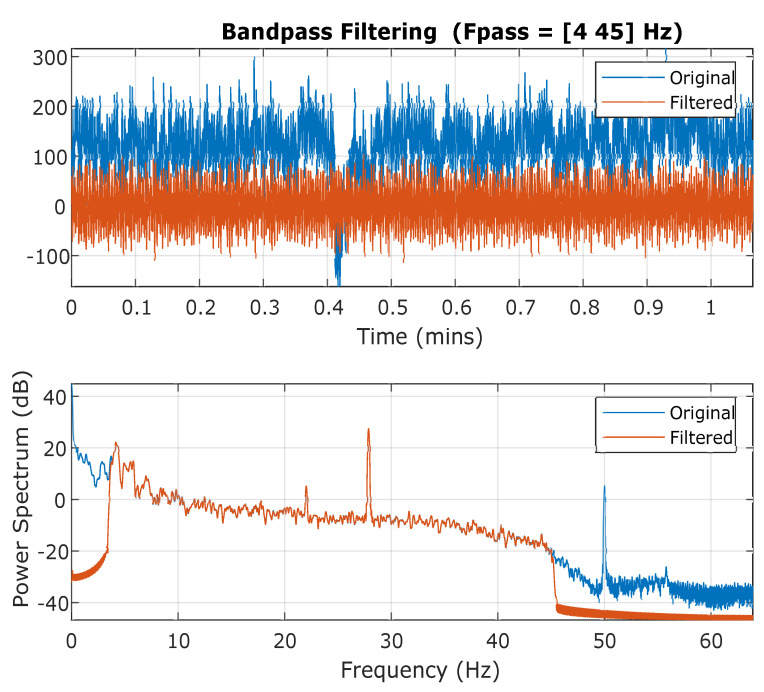
Band-pass filtering 4–45 Hz of individual EEG channel with power spectrum.

The second step of EEG pre-processing is band-pass filtering in which each channel of common referenced EEG signal is individually passed through band-pass filtering for noise removal. To compare against AMIGOS results, a passband frequency range of 4 Hz to 45 Hz is applied with transition band steepness of 0.85 and a stopband attenuation of 60 dB selected as a parameter. Figure 4 represents the original signal as an individual channel of EEG along with filtered signal after artifact removal using band-pass filtering. The effect of the band-pass filter is also illustrated as the power spectrum against frequency in Figure 4, where artifacts of below 4 Hz and around 50 Hz are removed from each EEG channel. After band-pass filtering, each channel of length 64 s is segmented by removing a 2 s signal from the start and 2 s of signal from the end to counter the effect of the filter at the edges. These three basic pre-processing steps result in EEG signals of length 60 s each (total of 7680 samples) for the DREAMER dataset. Therefore, the total size of DREAMER data for EEG after basic pre-processing is 5796 × 7680 (23 subjects, 18 trials, and 14 EEG channels).

#### 3.3.2. Basic ECG and GSR Pre-Processing

Peripheral signals of raw DREAMER data also undergo basic pre-processing steps to be consistent with the AMIGOS dataset. Therefore, both channels of ECG signals are individually down-sampled to 128 Hz from 256 Hz. After down-sampling, a low-pass filter of 60 Hz was applied to remove the high-frequency noise components from ECG data. After low-pass filtering, each channel of length 64 s is segmented by removing 2 s from start and 2 s of signal from the end to counter the effect of the filter at the edges. These three basic pre-processing steps result in ECG signals of length 60 s each (total of 7680 samples) for the DREAMER dataset. Therefore, the total size of DREAMER data for ECG after basic pre-processing is 828 × 7680 (23 subjects, 18 trials, and 2 ECG channels).

#### 3.3.3. Baseline Removal

Both DREAMER and AMIGOS datasets have baseline signals, where there is no stimulus provided to the subjects. AMIGOS has 5 s and DREAMER has 57 s of baseline signal recorded with no emotional activity. It is, therefore, useful to remove this neutral baseline activity from all the EEG, ECG, and GSR signals as a specific pre-processing step for emotion elicitation. For this purpose, baseline signals were divided into 1 s segments and then the mean of these segments is computed as the mean baseline activity of each signal as shown in Equation (3).

(3) $$meanBL=\frac{1}{S}\left(\sum_{s=1}^{S}BL_{s}\right)$$

EEG, ECG, and GSR signals data of 60sec emotional activity is also divided into 60 segments of 1 s each. After getting the mean of a specific channel for EEG, ECG, and GSR, each segment of emotional activity is subtracted from their corresponding mean segment of baseline activity to remove the neutral emotional effect as shown in Equation (4).

(4) $$blrSig_{s}=Sig_{s}-meanBL$$

**Figure 5:**
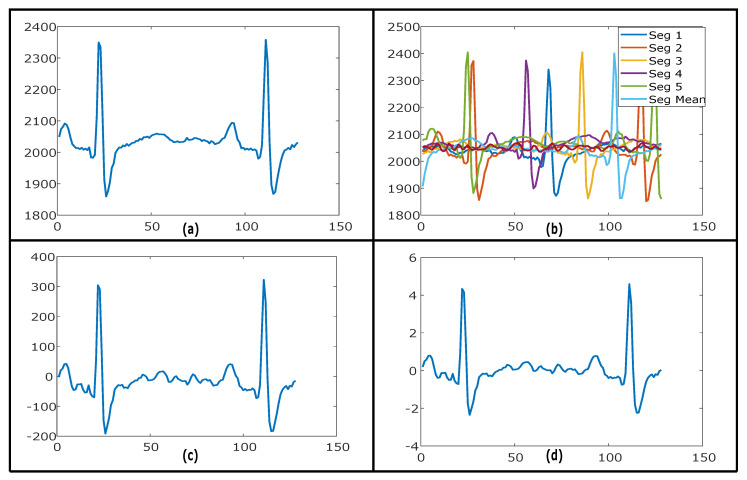
(**a**) ECG signal after basic pre-processing. (**b**) Five segments of ECG baseline activity with computed mean signal. (**c**) ECG signal (left channel) 1 s segment of emotional activity after removing mean of baseline activity. (**d**) After Z-score normalization.

These steps are performed to remove baseline from all the 14 EEG channels, 2 ECG channels, and from GSR to enhance and emphasize the emotional effect of corresponding stimuli. For the DREAMER dataset, the baseline was computed from the mean of all 57 segments of baseline activity. Figure 5a illustrates a segment of the ECG signal (Left Channel) of 1 s of emotional activity after basic pre-processing steps applied. Figure 5b represents five baseline segments of ECG with neutral activity and the computed mean of these five segments, while Figure 5c shows the output of baseline removal after subtracting segment of emotional activity represented in Figure 5a from the mean signal of five baseline segments represented in Figure 5b.

#### 3.3.4. Z-Score Normalization

Each of EEG, ECG, and GSR segments after baseline removal are normalized using Z-score normalization. Figure 5d illustrates the Z-score normalization of the already baseline removed ECG signal. This step is performed to prepare signals to use as a feature for neural network architecture after conversion to a common scale with unity standard deviation and zero mean. Both ECG channels and GSR are now prepared to use as a sequence input to deep neural network architecture for classification. Total size of ECG after pre-processing for DREAMER dataset is 828 × 60 × 128 (18 trials, 2 channels and 23 persons, 60 segments of 128 samples each) and for AMIGOS dataset is 1056 × 86.125 × 128 (16 trials, 2 channels and 33 persons, 86.125 average segments of 128 samples each). AMIGOS dataset has GSR with a total size of 528 × 86.125 × 128 after pre-processing.

#### 3.3.5. Preparation of EEG-Based 2D Images

Total size of EEG data after Z-score normalization for DREAMER dataset is 5796 × 60 × 128 (18 trials, 14 channels and 23 persons) and for AMIGOS dataset is 7392 × 86.125 × 128 (16 trials, 14 channels and 33 persons). Each example of EEG containing 14 channels of 128 samples each is mapped to Nasion 10–20 system [63] with their corresponding positioning of channels to a 1D topological vector of size 81. Each corresponding sample of 14 channels is mapped to 1D topological vector using the matching sequence of set of channels of AF3, F7, F3, FC5, T7, P7, O1, O2, P8, T8, FC6, F4, F8, AF4 to the set of indices of 4, 13, 19, 21, 29, 31, 37, 39, 47, 49, 55, 57, 67, 76 respectively while keeping all other indices of 1D topological vector as zero. After mapping, a feature matrix of 81 × 128 dimension is obtained as single EEG example of specific trial of a specific person. This feature matrix is now converted to a 2D image (PNG format) for further processing. After the preparation of 2D images, the DREAMER dataset contains 24,840 images and the AMIGOS dataset contains 45,474 images of 81 × 128 size each. These images are now ready to be used as input to the image input layer of deep neural network architecture.

### 3.4. Proposed DNN Classification Architectures

ECG and GSR modalities can be used as sequence input to DNN architecture, while 14 channels EEG after converted to 81 × 128 size of images can be used as image input to DNN architecture. Therefore two different DNN architectures were developed for classification through the neural network, one for EEG that would be useful for classification of images and another architecture for ECG and GSR, which would hypothetically be useful for classification of signals or time-series sequence data. Architecture for EEG primarily builds upon 2D convolutional layers, while the architecture for ECG and GSR primarily builds upon the combination of LSTM and 1D convolutional layers, as LSTM is expected to fully exploit the potential of sequence or time-series data [64]. The details of both approaches and the design of their deep network architectures are given below.

#### 3.4.1. DNN Architecture Design for EEG-Based Images

**Figure 6:**
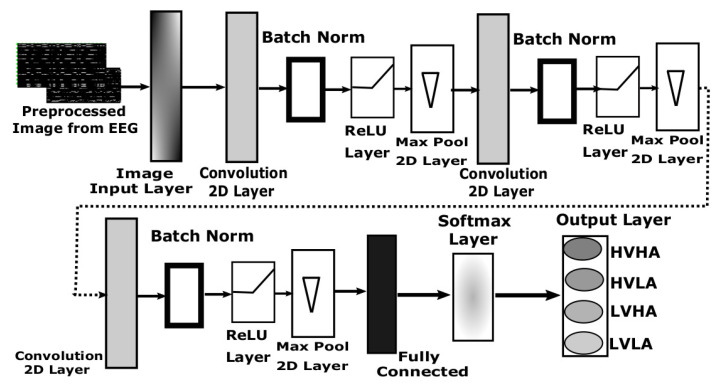
Design of deep neural network (2D-CNN) for EEG-based image data.

The detailed design of DNN architecture used for EEG image data is shown in Figure 6. This architecture accepts image data of size 81 × 128 in the image input layer. Then there are three sets of 2D convolutional layers, the first convolutional layer contains 8 filters of size 3 × 3, the second convolutional layer contains 16 filters of size 3 × 3 while third convolutional layer contains 32 filters of size 3 × 3 with padding of same values at the border. Each of these convolutional layers was followed by three layers of batch normalization, Rectified Linear Unit (ReLU) and 2D max-pooling layer. Each of the 2D max-pooling layers contains a pool size of 2 × 2 and a stride of 2 × 2 as well along with zero paddings. The third max-pooling layer is connected to a fully connected (FC) layer of size 4 which is then attached to the output layer after passing through the SoftMax layer. The findings from [65] suggest that for 2D-CNN architecture, wider datasets which have more number of classes require more FC layers as compared to deeper datasets which have fewer classes and more samples per class, and require fewer FC layers. Therefore, we used single FC layer in our 2D-CNN architecture.

The input image to this network is computed using a segment of 1 s of 14 channels of EEG with 128 samples. Therefore, the trained network requires only one second of EEG signals to classify into four basic emotions. This robust neural network has total learnable parameters of 80, 1168, 2320, and 43,012 for first, second, third convolution layers, and fully connected layer, respectively. The detailed parameters internal to the neural network architecture are represented in Table 1.

#### 3.4.2. DNN Architecture for ECG and GSR

**Figure 7:**
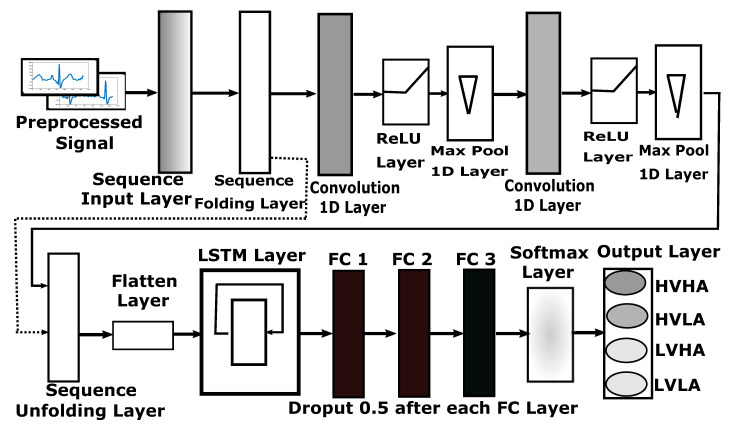
Design of deep neural network (1D-CNN + LSTM) for ECG and GSR-based sequence data.

Figure 7 present 1D-CNN architecture proposed for ECG and GSR modalities. One second signal from either ECG or GSR data is captured as a sequence. This sequence is then passed through two one-dimensional convolutional layers, each followed by ReLU activation and max-pooling layers to extract temporal features directly from time-series data. Extracted features are then flattened for the LSTM layer. This LSTM layer learns the order dependence between extracted temporal features, suitable for the classification of time-series data. Now three dense layers provide the learning of prediction probabilities from extracted features for four classes of emotion.

The detailed design of DNN architecture for ECG and GSR-based sequence data is illustrated in Figure 7. This architecture accepts sequence inputs of size 1 × 128 in its sequence input layer. After the sequence folding layer, two sets of 1D convolutional layers were added. First convolutional layer contains 16 filters of size 3 × 1, while second convolutional layer contains 32 filters of size 3 × 1. Each of these convolutional layers is followed by the ReLU layer and 1D max-pooling layer of size 2 × 1 with a stride of one. After max pooling, sequences are unfolded based on mini-batch size, and flatten layer was applied to get a feature vector. This feature vector is now passed through the LSTM layer with 128 hidden units, state activation function of tanh and gate activation function of sigmoid is used. LSTM layer is then followed by a series of three fully connected layers of size 256 for FC1, 128 for FC2, and a size of 4 for FC3. Each fully connected layer is followed by a dropout layer of 0.5, discarding 50% of random features to avoid over-training of sequence data from LSTM. The last FC layer after dropout is connected to the classification output layer through the SoftMax layer for the classification of HVHA, HVLA, LVHA, and LVLA classes of emotion.

Total learnable parameters of 160, 4640 and 2,163,200 exists for first convolution, second convolution and LSTM layer, respectively. Recurrent weights to train for LSTM are 512 × 128 with the 512 × 1 bias for T4096 vector size of input from flatten layer. The detailed parameters of activations, weights, bias, and learnables are provided in Table 2. Intermediate hidden-layer results are also represented in Section 5 for comparison of features using this deep neural network architecture.

### 3.5. Multi-Modal Fusion

To establish and compare the efficacy of the various combination of modalities, majority voting is applied. As every modality have their unique properties to depict the emotional state of a person, multi-modal fusion using majority voting at the decision level is used. This fusion helps to contribute comprehension of the effect of the various combination of modalities between GSR, ECG channels, and EEG.

## 4. Results

Pre-processed data for all modalities are now randomly split into 70% training data and 30% test data. After pre-processing, each modality contains 24,840 instances of data (81 × 128 size of images for EEG and 1 × 128 size of signals for ECG left, ECG right and GSR) for DREAMER dataset and 45,474 instances each for AMIGOS dataset. Two experiments were performed for each of AMIGOS and DREAMER datasets by randomly split into training and testing for the computation of results in both experiments. For the EEG approach, 13,642 images in the case of AMIGOS and 7452 images in the case of DREAMER were randomly selected as test data, while the remaining randomly selected images of these datasets were used as a training set of images. The same number of training and test samples respectively were randomly selected for ECG and GSR approaches as well.

For the computation of all the presented results, training parameters of deep neural network for both approaches of 2D-CNN and LSTM + 1D-CNN are consistent. Minimum batch size of 240, an initial learning rate of 0.001 with ADAM optimizer, and a gradient squared decay factor of 0.99 were used as training parameters. Core-i5 machine was used for the training of neural networks and testing of performance measures. Separate representation of results of both datasets is presented below.

### 4.1. AMIGOS Results

Table 3 illustrates the results computed for AMIGOS dataset. 2D-CNN-based approach was used to train 31,832 images randomly selected from the dataset. A similar number of instances from ECG left channel, ECG right channel, and GSR was incorporated to individually train LSTM + 1D-CNN-based neural network. ECG right channel among this second approach depicts the highest accuracy of 98.73%, while GSR with the lowest average accuracy of 63.67%. EEG signal using a 2D-CNN-based approach depicts quite low performance 74.65% as compared to ECG, but much better than the existing approaches in the literature for AMIGOS.

Table 3 depicts the better performance of the ECG right channel as compared to ECG left channel. Similarly, both of ECG channels performed better as compared to EEG modality, while EEG modality performs better than GSR. We used 2D-CNN architecture for EEG modality, while the same 1D-CNN architecture is used for both ECG channels and GSR modality. The relatively low performance of EEG is because of different architecture used for its 14 channel combinations, while the relatively low performance of GSR is because of the nature of its modality. GSR generally depicts less contribution in the evaluation of emotion elicitation as compared to ECG [66], which is enriched with more critical information regarding the emotional state of a person. The fusion of ECG left channel, ECG right channel, and EEG channel improve overall highest classification accuracy of 99%. The results for all combinations of the modalities are separately represented in Table 4.

### 4.2. DREAMER Results

Table 5 illustrates the summary of results computed for two random splits for ECG channel 1, channel 2, and EEG. 2D-CNN network was trained with 17,388 randomly selected images and a similar number of samples from ECG channel 1 and ECG channel 2 were used to individually train the LSTM + 1D-CNN-based neural network. ECG channel 2 results in the highest average accuracy among these modalities, while EEG with another approach has low but comparable significant results for the classification of four classes in the literature. Table 6 represents the results of majority voting using combinations of channel 1 of ECG, channel of ECG, and EEG.

The detailed results of the DREAMER dataset can be evaluated using confusion matrices for individual modalities as well as the fusion of modalities. Therefore, Table 7 and Table 8 represents confusion matrices of ECG channel 1 and channel 2 respectively. Both channels of ECG depict promising results as compared to EEG, while Table 9 represents detailed results of EEG modality. Similarly, the detailed results of AMIGOS dataset in terms of confusion matrices are presented in Table 10 for ECG right-channel modality, Table 11 for GSR modality, and Table 12 for EEG modality respectively.

In both datasets, the recognition rate of ECG is better than EEG because of the difference in architecture. The combination of ECG and EEG yields better performance for both datasets because these two are the highest achieving modalities individually. Secondly, architecture for ECG and EEG is different, therefore EEG with relatively lower individual accuracy performed well on samples converted into images for 2D-CNN architecture. The architecture used for both modalities of ECG and GSR is the same, therefore the small accuracy value of individual GSR modality is due to less informative nature of GSR signals as compared to ECG signals, which results in deterioration in overall recognition rate when combined with ECG and EEG. In the DREAMER dataset, GSR signals are not available, therefore, GSR is not combined for the representation of that dataset results.

## 5. Discussion

After results analysis, it is observed that AMIGOS performed better as compared to DREAMER dataset with the same methodology. The second observation from Table 9 and Table 10 exhibits that the response of all the four classes of HVHA, HVLA, LVHA, and LVLA is consistent for a specific modality, except a significantly better response of LVHA in case of GSR modality as shown in Table 11. The third observation is that the response of these four classes is also consistent for the specific modality of the DREAMER dataset as shown in Table 7, Table 8 and Table 9, but based on the contribution of each class in the overall dataset. This could also point towards the observation of more imbalance class instances for the DREAMER dataset as compared to the AMIGOS dataset.

Based on these above-mentioned observations, one possible reason for AMIGOS outperforming the DREAMER dataset is its more balanced distribution of classes as compared to DREAMER. Another reason for the better performance of AMIGOS as compared to DREAMER is due to the nature of the self-assessment acquisition process. Self-assessment for the AMIGOS dataset was obtained on a scale of 1–9 for arousal and valence separately. However, for the DREAMER dataset, self-assessment from subjects was acquired on the scale of 1–5 for both valence and arousal. The scale of 1–5 not only exhibits half the freedom of choice on an intensity scale of emotion but also restricts the imbalance created by avoiding the midpoint between 1–5 scale as participants can only provide integer data for the intensity of arousal and valence. However, AMIGOS gives participants the liberty to self-assess in a floating-point number for the scale of 1–9, hence better categorization of emotion can be made which implied better performance of the algorithm on this dataset comparatively. One more possible reason for variation in results is because the total number of instances in AMIGOS for the specific trial is variable and based on the actual length of the trial; however, in the case of DREAMER, only the last 60 segments (1 s each) of each trial were incorporated (because a few trials are much larger in length to be significant for specific emotion). As AMIGOS explores the full-length potential of each trial, this could also explain the variation between the performance of AMIGOS and the DREAMER dataset.

**Figure 8:**
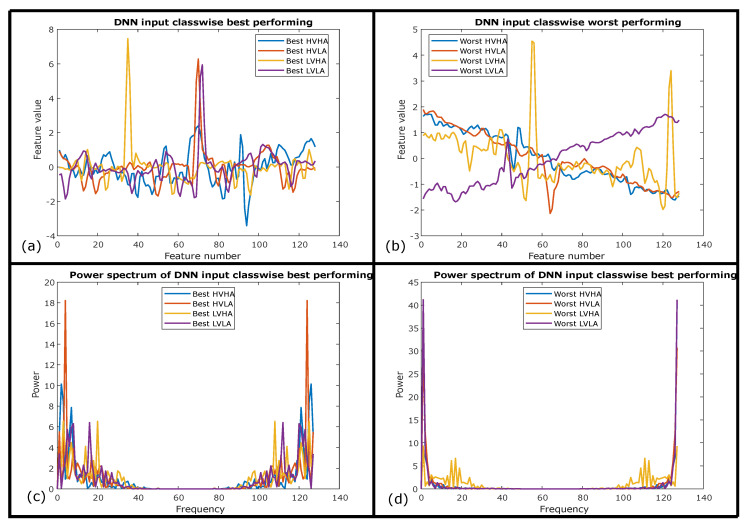
(**a**) Best-performing class-wise ECG samples. (**b**) Worst-performing class-wise ECG samples. (**c**) Spectrum of best-performing class-wise ECG samples. (**d**) Spectrum of worst-performing class-wise ECG samples.

It is also interesting to investigate the spectral signature of ECG signals and compared it with GSR signals to elaborate on the significance of ECG results as compared to GSR results for the same deep neural network architecture. Best and worst-performing ECG instances from test data of AMIGOS, based on their prediction probabilities were selected for four classes individually are presented in Figure 8a,b respectively. Signals of all classes except LVHA are baseline shifted with high noise content hiding the actual shape of an ECG signal results in its worst performance for the use of emotion elicitation. The power spectrum comparison for four classes of best and worst of these signals is presented in Figure 8c,d, respectively. Figure 8c depicts higher inter-class spectral variability of best-performing signals as compared to lower inter-class spectral variability of worst signals except for LVHA in Figure 8d.

**Figure 9:**
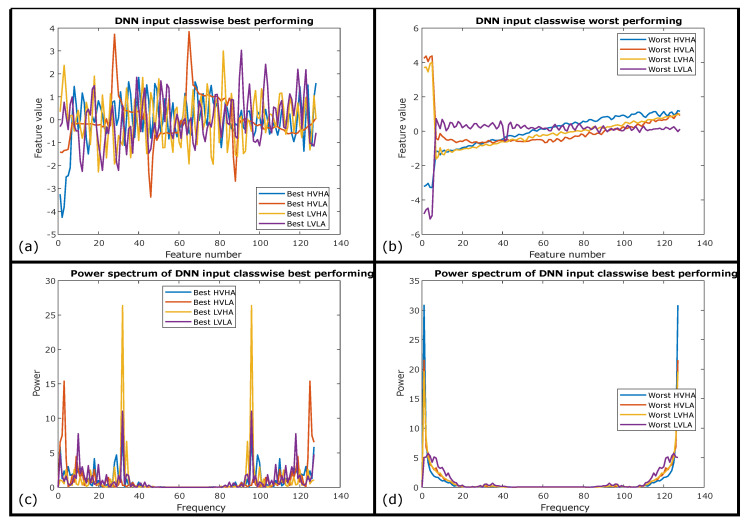
(**a**) Best-performing class-wise GSR samples. (**b**) Worst-performing class-wise GSR samples. (**c**) Spectrum of best-performing class-wise GSR samples. (**d**) Spectrum of worst-performing class-wise GSR samples.

A strong connection between the performance of proposed deep neural network architecture and spectral inter-class variability can also be proven through spectral analysis of comparatively less significant modality of GSR. Therefore, Figure 9a,c exhibits higher inter-class temporal and spectral variability of best-performing GSR signals as compared to lower inter-class temporal and spectral variability of worst-performing GSR signals as shown in Figure 9b,d respectively with the exception of LVHA class. This exception of LVHA class depicts higher recognition performance for LVHA which is also evident from confusion matrices of AMIGOS dataset presented in Table 10 and Table 11 for both ECG and GSR respectively. Another observation can be drawn with the higher inter-class variability of the ECG signal in Figure 8c as compared to the GSR signal in Figure 8, representing the spectral significance for the emotion elicitation process as a performance comparison of these two modalities. Therefore, these temporal and spectral observations proved the significance of proposed CNN + LSTM-based architecture to extract those temporal-spectral features for emotion elicitation.

**Figure 10:**
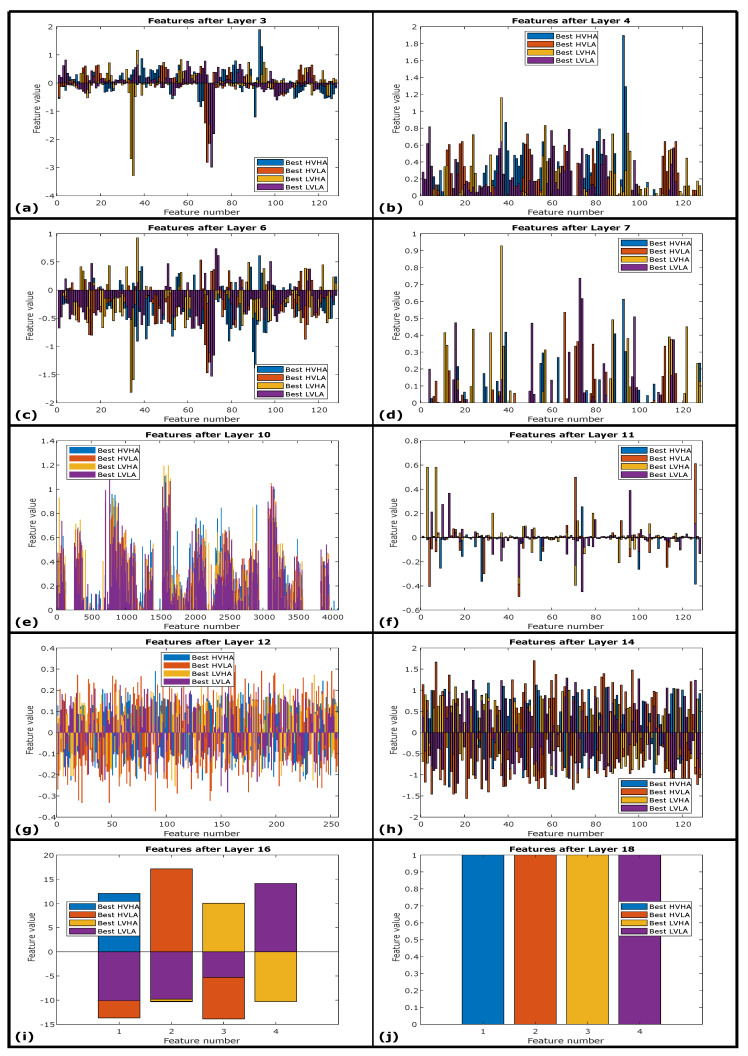
(**a**) Best class-wise features after first convolution layer. (**b**) Best class-wise features after first ReLU layer. (**c**) Best class-wise features after second convolution layer. (**d**) Best class-wise features after second ReLU layer. (**e**) Best class-wise features after flatten layer. (**f**) Best class-wise features after LSTM layer. (**g**) Best class-wise features after FC1 layer. (**h**) Best class-wise features after FC2 layer. (**i**) Best class-wise features after FC3 layer. (**j**) Best class-wise predictions after SoftMax layer.

**Figure 11:**
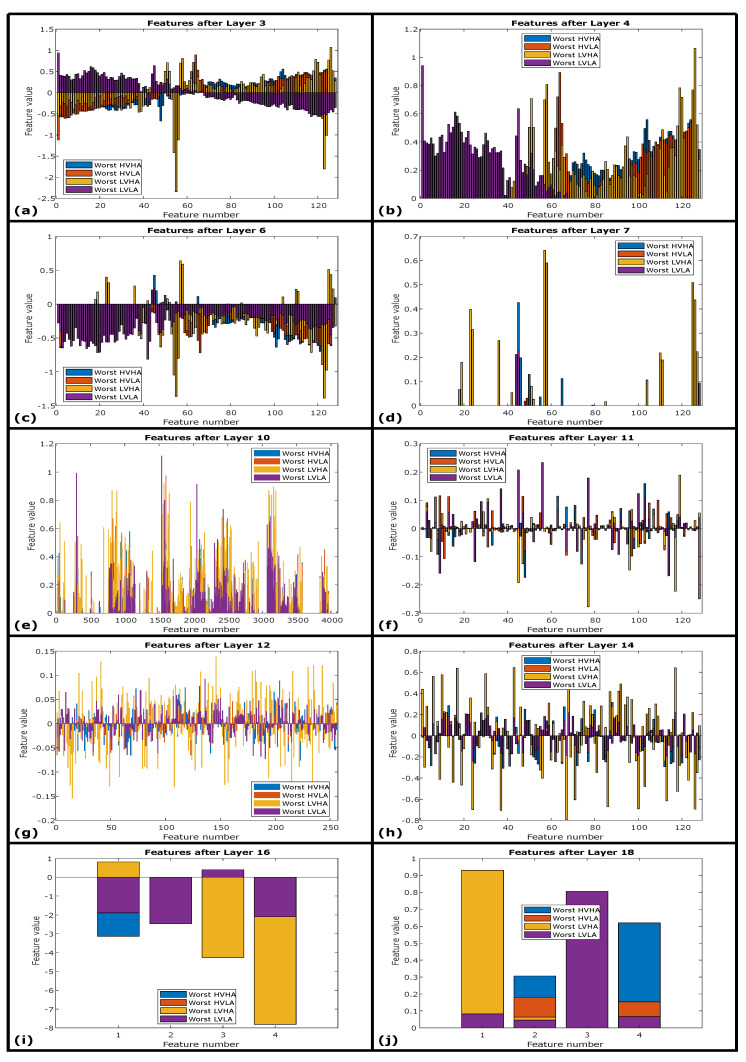
(**a**) Worst class-wise features after first convolution layer. (**b**) Worst class-wise features after first ReLU layer. (**c**) Worst class-wise features after second convolution layer. (**d**) Worst class-wise features after second ReLU layer. (**e**) Worst class-wise features after flatten layer. (**f**) Worst class-wise features after LSTM layer. (**g**) Worst class-wise features after FC1 layer. (**h**) Worst class-wise features after FC2 layer. (**i**) Worst class-wise features after FC3 layer. (**j**) Worst class-wise predictions after SoftMax layer.

Intermediate features from hidden layers of 1D-CNN + LSTM architecture are also interesting to investigate for best-performing features in Figure 10 and worst-performing features in Figure 11 respectively. These layers’ wise deep features are the continuity of input signals of best-performing ECG instances and worst-performing ECG instances represented in Figure 8a,b respectively. Figure 10a,c represents extracted features after first and second convolution layers of 1D-CNN + LSTM architecture while Figure 10b,d represents extracted features after first and second hidden ReLU layers respectively. These hidden deep features from best ECG instances are about to evolve in next hidden layers, where the flatten layer converts 32 instances of activations of 128 samples each into 4096 size feature vector as shown in Figure 10e. This feature vector is passed through the LSTM layer with feature representation in Figure 10f and with feature representation of first, second, and third fully connected layers shown in Figure 10g–i respectively. Through all these hidden layers features evolve with the improvement in inter-class variability in the subsequent layers.

Figure 11a–h also describe the hidden feature representations evolved through subsequent layers for worst ECG instances for the same 1D-CNN + LSTM architecture. It can be observed that the inter-class variability improves significantly after LSTM and for deep layers of architectures such as represented in Figure 11g,h. However, it is also evident that the overall inter-class variability enhancement for emotion classification is much smaller as compared to the improvement found for best-performing ECG instances. Signals performed worst primarily since these signals are inherited with strong input noise, which is unable to be removed through pre-processing steps performed before introducing the signals to DNN.

This investigation can be evaluated with the importance of a strong linkage between spectral inter-class variability and the performance of proposed deep neural network architecture. The better response of the LVHA class for both ECG and GSR modalities is strongly linked with its higher spectral inter-class variability for the AMIGOS dataset as highlighted in Table 10 and Table 11. Therefore, besides the significance of LVHA class, it is wrongly classified as HVHA and other wrong decisions represented in Figure 11i,j. The best-performing signals are predicted with the highest prediction probabilities are represented in Figure 10i,j for FC3 and SoftMax layer respectively. It can also be concluded that the 1D-CNN + LSTM-based deep architecture is highly capable of extracting and distinguishing spectral features that support better performance of the architecture.

The main objective of this study is to improve recognition performance of four classes of emotions in a less constrained real-world environments. To evaluate the accomplishment of this objective, we need to explore the performance comparison of this study with previous literature using benchmark datasets. This comparison is made on the two publicly available datasets of AMIGOS and DREAMER. Physiological signals acquired for both of these recently published DREAMER and AMIGOS datasets use wearable low-cost sensors for EEG, ECG and GSR acquisition as compared to invasive acquisition of physiological signals in previously published DEAP and MAHNOB-HCI datasets. Therefore, comparison of emotion recognition of state of the art with proposed study for both datasets is presented in Table 13.

In Table 13, two classes of emotions represent either high/low levels of valence or arousal, while four classes represent HVHA, HVLA, LVHA, and LVLA. For instance, the study [36] with statistical features and SVM using AMIGOS dataset results in 68.8% and 67% of accuracy for arousal and valence respectively, while another study [37] improved valence results by using SVM-RBF. The recognition results from [12,13,35,39] and attention-based LSTM-RNN study [40] improved with deep-learning algorithms for either AMIGOS or DREAMER dataset for two classes of emotions. In a recent study using Bayesian DNN [42], only binary classification of high and low level of valence results are reported as 86% for DREAMER dataset and 90% for AMIGOS dataset. 3D-CNN architecture [14] yields good results for AMIGOS dataset with the cost of computational complexity and expensive training time and resources. In a more recent study [15], four classes of emotions with recognition performance of 55.56% and 58.57% is achieved using transfer learning of CNN-VGG16 model [67] for DREAMER and AMIGOS datasets respectively. The combination of 1D-CNN and LSTM used to develop a robust methodology that performed much better than the previous results for two of the latest publicly available datasets suitable for real-world emotion monitoring. Table 13 summarized the comparison between proposed methodology with state-of-the-art research using physiological-based emotion elicitation.

Therefore, our proposed architecture outperformed state-of-the-art approaches for the physiological signals acquired through less invasive methods. ECG proved to be a more significant modality as compared to EEG and GSR through the design of specialized deep neural networks based on the nature of the physiological signals. Spectral analysis with a performance comparison for both datasets appraises the adequacy of our methodology to inherently extract spectral features from these modalities as well.

## 6. Conclusions

Our proposed deep-learning architecture combines the usefulness of LSTM and CNN, which proved to be efficient for emotion recognition and outperforming previous approaches. Intermediate results of the deep neural network as a hidden feature representation helps us get an insight into features evolving through these layers. Furthermore, the significance of the proposed methodology lies in the higher performance for four classes of emotion elicitation based on subject-independent study, while the wireless acquisition of physiological signals is more suitable for the less constrained real-world environments.

The scope of this study is limited to the decision-level fusion of modalities using majority voting; however, feature-level fusion techniques with proposed architecture may yield significant results. Similarly, three-dimensional CNN architectures can be explored with the combination of LSTM as future investigation for performance improvement. Future dimensions will also assist EEG with more specialized deep neural networks for better performance as well for various other physiological signals such as skin temperature and respiration.

## Figures and Tables

**Table 1:** Details of 2D-CNN Architecture.

Serial	Layer Type	Activations	Weights/Offset	Bias/Scale	Learnables
1	Image Input	81 × 128 × 1	-	-	0
2	Convolution	81 × 128 × 1	3 × 3 × 1 × 8	1 × 1 × 8	80
3	Batch Normalization	81 × 128 × 1	1 × 1 × 8	1 × 1 × 8	16
4	ReLU	81 × 128 × 1	-	-	0
5	Ma × Pooling	41 × 64 × 8	-	-	0
6	Convolution	41 × 64 × 16	3 × 3 × 8 × 16	1 × 1 × 16	1168
7	Batch Normalization	41 × 64 × 16	1 × 1 × 16	1 × 1 × 16	32
8	ReLU	41 × 64 × 16	-	-	0
9	Ma × Pooling	21 × 32 × 16	-	-	0
10	Convolution	21 × 32 × 16	3 × 3 × 16 × 16	1 × 1 × 16	2320
11	Batch Normalization	21 × 32 × 16	1 × 1 × 16	1 × 1 × 16	32
12	ReLU	21 × 32 × 16	-	-	0
13	Fully Connected	1 × 1 × 4	4 × 10,752	4 × 1	43,012
14	Softma×	1 × 1 × 4	-	-	0
15	Classification Output	-	-	-	0

**Table 2:** Details of 1D-CNN + LSTM Architecture.

Serial	Layer Type	Activations	Weights	Bias	Learnables
1	Sequence Input	128 × 1 × 1	-	-	0
2	Sequence Folding	128 × 1 × 1	-	-	0
3	Convolution	128 × 1 × 16	3 × 1 × 1 × 16	1 × 1 × 16	64
4	ReLU	128 × 1 × 16	-	-	0
5	Ma × Pooling	128 × 1 × 16	-	-	0
6	Convolution	128 × 1 × 32	3 × 1 × 16 × 32	1 × 1 × 32	1568
7	ReLU	128 × 1 × 32	-	-	0
8	Ma × Pooling	128 × 1 × 32	-	-	0
9	Sequence Unfolding	128 × 1 × 32	-	-	0
10	Flatten	4096	-	-	0
11	LSTM	128	Input: 512 × 4096, Recurrent: 512 × 128	512 × 1	2,163,200
12	Fully Connected	256	256 × 128	256 × 1	33,024
13	Dropout	256	-	-	0
14	Fully Connected	128	128 × 256	128 × 1	32,896
15	Dropout	128	-	0
16	Fully Connected	4	4 × 128	4 × 1	516
17	Dropout	4	-	-	0
18	Softma×	4	-	-	0
19	Classification Output	-	-	-	0

**Table 3:** Summary of results for AMIGOS dataset. Highlighted row represents best-performing modality.

Modality	1st Random Split	2nd Random Split	Average Accuracy
ECG (Left)	96.98%	96.93%	96.96%
**ECG (Right)**	**98.81%**	**98.65%**	**98.73%**
GSR	63.77%	63.56%	63.67%
EEG	76.39%	72.91%	74.65%

**Table 4:** AMIGOS results for majority voting (fusion of modalities). Highlighted row represents the best-performing combination of modalities.

Modality	1st Random Split	2nd Random Split	Average Accuracy
ECGL + ECGR + GSR + EEG	97.5%	97.0%	97.25%
ECGL + ECGR	98.2%	97.7%	97.95%
ECGL + GSR	79.8%	81.6%	80.70%
ECGL + EEG	85.4%	78.9%	82.15%
ECGR + GSR	80.5%	82.5%	81.50%
ECGR + EEG	86.1%	79.5%	82.80%
GSR + EEG	68.7%	65.8%	67.25%
ECGL + ECGR + GSR	98.5%	98.2%	98.35%
**ECGL + ECGR + EEG**	**99.0%**	**98.6%**	**98.8%**
ECGL + GSR + EEG	91.5%	90.0%	90.75%
ECGR + GSR + EEG	92.2%	90.5%	91.35%

**Table 5:** Summary results for DREAMER dataset. Highlighted row represents best-performing modality.

Modality	1st Random Split	2nd Random Split	Average Accuracy
ECG (Channel 1)	90.46%	88.31%	89.39%
**ECG (Channel 2)**	**90.03%**	**90.96%**	**90.50%**
EEG	47.44%	49.64%	48.54%

**Table 6:** DREAMER results for majority voting. Highlighted row represents best-performing combination of modalities.

Modality	1st Random Split	2nd Random Split	Average Accuracy
ECGL + ECGR	89.5%	89%	89.25%
ECGL + EEG	63.9%	61.6%	62.75%
ECGR + EEG	63.9%	62.2%	63.05%
**ECGL + ECGR + EEG**	**90.8%**	**90.3%**	**90.55%**

**Table 7:** Confusion matrix of ECG channel 1 of DREAMER dataset.

	Target Class					
		HVHA	HVLA	LVHA	LVLA	Total
**Output** **Class**	**HVHA**	2932 39.3%	69 0.9%	143 1.9%	60 0.8%	91.5%
	**HVLA**	88 1.2%	1266 17%	69 0.9%	29 0.4%	87.2%
	**LVHA**	121 1.6%	48 0.6%	2010 27%	47 0.6%	90.3%
	**LVLA**	27 0.4%	3 0.0%	10 0.1%	530 7.1%	93.0%
	**Total**	92.6%	91.3%	90.1%	79.6%	90.4%

**Table 8:** Confusion matrix of ECG channel 2 of DREAMER dataset.

	Target Class					
		HVHA	HVLA	LVHA	LVLA	Total
**Output** **Class**	**HVHA**	2926 39.3%	84 1.1%	138 1.9%	61 0.8%	91.2%
	**HVLA**	78 1.0%	1221 16.4%	47 0.6%	39 0.5%	88.2%
	**LVHA**	155 2.1%	76 1.0%	2037 27.3%	45 0.6%	88.1%
	**LVLA**	9 0.1%	5 0.1%	10 0.1%	521 7.0%	95.6%
	**Total**	92.4%	88.1%	91.3%	78.2%	90.0%

**Table 9:** Confusion matrix of EEG of DREAMER dataset.

	Target Class					
		HVHA	HVLA	LVHA	LVLA	Total
**Output** **Class**	**HVHA**	2142 28.7%	656 8.8%	1037 13.9%	256 3.4%	52.4%
	**HVLA**	315 4.2%	397 5.3%	273 3.7%	82 1.1%	37.2%
	**LVHA**	490 6.6%	223 3.0%	784 10.5%	116 1.6%	48.6%
	**LVLA**	221 3.0%	110 1.5%	138 1.9%	212 2.8%	31.1%
	**Total**	67.6%	28.6%	35.1%	31.8%	47.4%

**Table 10:** Confusion matrix of ECG right modality for AMIGOS dataset.

	Target Class					
		HVHA	HVLA	LVHA	LVLA	Total
**Output** **Class**	**HVHA**	3027 22.2%	14 0.1%	13 0.1%	8 0.1%	98.9%
	**HVLA**	13 0.1%	3139 23.0%	10 0.1%	9 0.1%	99.0%
	**LVHA**	14 0.1%	19 0.1%	3664 26.9%	22 0.2%	98.5%
	**LVLA**	20 0.1%	10 0.1%	11 0.1%	3649 26.7%	98.9%
	**Total**	98.5%	98.6%	99.1%	98.9%	98.8%

**Table 11:** Confusion matrix of GSR modality for AMIGOS dataset.

	Target Class					
		HVHA	HVLA	LVHA	LVLA	Total
**Output** **Class**	**HVHA**	1950 14.3%	332 2.4%	313 2.3%	444 3.3%	64.2%
	**HVLA**	386 2.8%	1958 14.4%	380 2.8%	495 3.6%	60.8%
	**LVHA**	420 3.1%	530 3.9%	2656 19.5%	617 4.5%	62.9%
	**LVLA**	318 2.3%	362 2.7%	349 2.6%	2132 15.6%	67.4%
	**Total**	63.4%	61.5%	71.8%	57.8%	63.7%

**Table 12:** Confusion matrix of EEG modality for AMIGOS dataset.

	Target Class					
		HVHA	HVLA	LVHA	LVLA	Total
**Output** **Class**	**HVHA**	2356 17.3%	282 2.1%	321 2.4%	287 2.1%	72.6%
	**HVLA**	293 2.1%	2429 17.8%	350 2.6%	299 2.2%	72.1%
	**LVHA**	231 1.7%	276 2.0%	2819 20.7%	285 2.1%	78.1%
	**LVLA**	194 1.4%	195 1.4%	208 1.5%	2817 20.6%	82.5%
	**Total**	76.6%	76.3%	76.2%	76.4%	76.4%

**Table 13:** Comparison with state-of-the-art related work. V and A represents binary classification (High or Low) of Valence and Arousal respectively, while 4 classes are HVHA, HVLA, LVHA and LVLA.

Study	Methodology	Emotion Classes	Accuracy (DREAMER)	Accuracy (AMIGOS)
[36]	Statistical Features, SVM	2	-	67%(V), 68.8%(A)
[37]	SVM-RBF	2	-	83.9%(V), 65%(A)
[35]	Statistical Features, SVM	2	61.84%(V), 63.32%(A)	-
[12]	DGCNN	2	86.23%(V), 84.54%(A)	-
[13]	GCB-net	2	86.99%(V), 89.32%(A)	-
[39]	CNN	2	-	84%(V), 85.8%(A)
[40]	LSTM-RNN	2	-	79.4%(V), 83.3%(A)
[14]	3D-CNN	4	-	99.7%
[42]	Bayesian DNN	2	86%(V)	90%(V)
[15]	CNN-VGG16	4	55.56%	58.57%
Proposed Study	LSTM + CNN	4	90.8%	99%
